# Sidedness is prognostic in locoregional colon cancer: an analysis of 9509 Australian patients

**DOI:** 10.1186/s12885-017-3255-z

**Published:** 2017-04-08

**Authors:** Daniel Brungs, Morteza Aghmesheh, Paul de Souza, Weng Ng, Wei Chua, Martin Carolan, Philip Clingan, Emma Healey, June Rose, Tameika Tubaro, Marie Ranson

**Affiliations:** 1 Illawarra Health and Medical Research Institute, University of Wollongong, Wollongong, NSW Australia; 2 School of Biological Sciences, University of Wollongong, Wollongong, NSW Australia; 3 Illawarra Cancer Centre, Wollongong Hospital, Wollongong, NSW Australia; 4 CONCERT-Translational Cancer Research Centre, Sydney, NSW Australia; 5 Medical Oncology Department, Liverpool Hospital, Sydney, NSW Australia; 6 Ingham Institute for Applied Medical Research, Liverpool Hospital, Sydney, NSW Australia; 7 School of Medicine, Western Sydney University, Sydney, NSW Australia; 8 South Western Medical School, University of New South Wales, Sydney, NSW Australia

**Keywords:** Colonic neoplasms/mortality, Colonic neoplasms/pathology, Neoplasm staging

## Abstract

**Background/Aim:**

Right sided colon cancer (RsCC) is proposed to be a distinct disease entity to left sided colon cancer (LsCC). We seek to confirm primary tumour location as an independent prognostic factor in locoregional colorectal cancer.

**Methods:**

All patients with stage I – III primary adenocarcinoma of colon were identified from the New South Wales (NSW) clinical cancer registry (2006–2013). Primary tumour location (RsCC vs LsCC) survival analyses were conducted using the Kaplan-Meier method, and adjusted hazard ratios for 5-year all-cause mortality (OS) and 5-year cancer specific mortality (CSS) were obtained using Cox proportional hazards regression.

**Results:**

We identified 9509 patients including 5051 patients with RsCC and 4458 with LsCC. Patients with RsCC were more likely to be older, female, have a higher Charlson comorbidity index, and have worse tumour prognostic factors. In univariate analysis of all stages combined, those patients with RsCC had a worse overall survival (OS, HR 1.20 95% CI 1.11–1.29, *p* < 0.0001), although this was not significant in the multivariate analysis (HR 0.96 95% CI 0.89–1.04, *p* = 0.35). Stage I patients with RsCC had a trend to improved OS (multivariate HR 0.84 95% CI 0.69–1.01, *p* = 0.07) and a significantly improved CSS (multivariate HR 0.51 95% CI 0.35–0.75, *p* = 0.0006). In stage II patients with RsCC there was a significantly improved OS (multivariate HR 0.85 95% CI 0.75–0.98, *p* = 0.02) and CSS (multivariate HR 0.59 95% CI 0.45–0.78, *p* = 0.0002) compared to LsCC. In stage III patients, those with RsCC had a worse OS (multivariate HR 1.13 95% CI 1.01–1.26, *p* = 0.032) and a trend to worse CSS (multivariate HR 1.12 95% CI 0.94–1.33, *p* = 0.22).

**Conclusions:**

Primary tumour location is an important prognostic factor in locoregional colon cancer with an effect that varies by stage. RsCC is associated with lower all-cause mortality in stage II, and higher all-cause mortality in stage III.

**Electronic supplementary material:**

The online version of this article (doi:10.1186/s12885-017-3255-z) contains supplementary material, which is available to authorized users.

## Background

Colorectal (CRC) is a common and lethal malignancy, projected to account for 13% of all new cancer cases diagnosed in Australia in 2015, and 10% of Australian cancer deaths [1]. In recent years there has been increasing interest in identifying the differences between right sided and left sided colon cancer, and the potential for using this clinical marker as a surrogate marker of tumour biology, with the intent of improved personalisation of systemic treatments.

There is a growing body of evidence to suggest that right sided colon cancers (RsCC) follow a different disease process compared to left sided tumours (LsCC). The proximal and distal colons are physiologically separate, arising from distinct embryological origins, with differences in tumour genetics, histology, presentation, and clinical features [2–4]. Patients with RsCC are older, more likely to be female, have more comorbidities, with poorer tumour histopathological features [5–8].

Despite this, there is ongoing debate whether primary tumour location is an independent prognostic factor in colon cancer. Most, but not all studies have found poorer survival with RsCC [7–11]. A recent meta-analysis found a statistically significant worse overall survival in patients with RsCC, although there was significant heterogeneity seen due the spectrum of included study designs, disease stage, and limited information about treatment received by patients [12]. Tumour stage may play a role, with a large Surveillance, Epidemiology, and End Results (SEER) program study showing worse overall survival in Stage III RsCC patients, but not in Stage I or II [7], although these finding have been recently challenged by a propensity score matched analysis of the SEER database, which showed a better prognosis in RsCC patients [9].

This current study aims to use a prospectively collected database of Australian patients to determine whether primary tumour location is an independent prognostic factor in locoregional colon cancer, and compare our findings to the literature.

## Methods

### Patient cohort

The New South Wales (NSW) clinical cancer registry contains demographic and clinical data for patients diagnosed or treated for cancer in NSW, covering approximately 30% of the Australian population. Data is collected from pathological laboratories, hospitals and oncology departments under mandatory notification of new cancer cases irrespective of treatment.

We identified all patients with Stage I, II or III colorectal cancer in NSW from Jan 2006 to 2013 (*n* = 9509) as per third edition of the International Classification of Diseases for Oncology (ICD-O-3) [13]. The registry also contained adjuvant chemotherapy treatment details for a more limited group of patients with stage II and III disease (*n* = 4102).

Mortality data, including cause of death, was obtained with linkage to the NSW registry of Births, Deaths and Marriages (BDM) by the Centre for Health Record Linkage (CHeReL) [14]. The censor data for survival data was 1st December 2014. Primary tumour location was defined right sided (caecum to transverse colon) or left sided (splenic flexure to rectosigmoid). Patients with rectal cancer were excluded from analysis due to the different treatment paradigm to colon cancer in locoregional disease. No data was available for cause of death in 935 patients (10.1%) which were therefore excluded from the cancer specific death analyses. Patients were deemed to have died as a result of colon cancer only if the underlying cause of death, rather than an associated cause of death, was coded as C18–20.

Comorbidity data was obtained by CHeReL linkage of the clinical cancer registry data to the Admitted Patient Data Collection (APDC). The APDC contains all admitted patient services provided by New South Wales Public Hospitals, Public Psychiatric Hospitals, Public Multi-Purpose Services, Private Hospitals, and Private Day Procedures Centres. Comorbidities of each patient were quantified using the Charlson comorbidity index which predicts mortality from a range of 22 comorbid conditions [16]. ICD-10 codes were extracted from admissions prior to diagnosis, then translated into a Charlson comorbidity index (modified for cancer) using methods previously described [15, 16].

All data linkage was performed by the Centre for Health Record Linkage, with only de-identified information provided to the researchers. The data sources used for this study required ethical and data custodian approval to access, link (by an independent and approved authority) and release for research. Approval for this project was provided by the NSW Population & Health Services Research Ethics Committee (approval HREC/13/CIPHS/39).

### Statistical analysis

Our primary outcome was all-cause 5-year overall survival (OS) stratified by stage, defined as death within 5 years of primary diagnosis of colon cancer on basis of dates recorded in the cancer registry and BDM databases. The secondary outcome was cancer specific 5 year survival (CSS) stratified by stage, as per cause of death encoded on BDM data. Median values for OS and CSS-OS and corresponding 95% CI were calculated using Kaplan-Meier methods. Unadjusted and multivariable Cox proportional hazards regression analyses were used to estimate the association between tumour location and survival and to calculate corresponding hazard ratios (HRs) and 95% confidence intervals (CIs). The following variables were included in the multivariate model: age, sex, Charlson Comorbidity Index, TNM stage, year of diagnosis, grade, and adjuvant treatment (receipt and type of adjuvant treatment performed in subset of patients only). All statistical analyses were performed using SAS 9.2 software (SAS Institute, Inc., Cary, NC).

## Results

### Patient characteristics (*n = 9509)*

The characteristics of the NSW cohort is summarised in Table 1. The mean follow up was 46 months (interquartile range 27 to 71 months). At the end of 5 years of follow up, 2686 (28.2%) patients had died, with 913 reported deaths (34.0% of deaths) due to colon cancer. 22% of patients had stage I disease, 39% stage II, and 39% had Stage III. There were slightly more RsCC (53%) than LsCC (47%). Patients with RsCC were older (61% vs 47% older than 70 years), more likely to be female (54% vs 42% female), had higher Charlson comorbidity indices (CCI, 40% vs 34% CCI ≥ 1), and had worse prognostic features including higher TNM stage (79% vs 76% stage II/III), and higher grade tumour (23% vs 11% poorly differentiated).

**Table 1:** Patient characteristics (*n* = 9509)

Characteristic		All Patients (%)	Right sided tumour (%)	Left sided tumour (%)	*P* value
TNM stage	I	2104 (22)	1055 (21)	1049 (24)	<0.0001
	II	3684 (39)	2059 (41)	1625 (36)	
	III	3721 (39)	1937 (38)	1784 (40)	
T stage	1	1526 (16)	715 (14)	811 (18)	<0.0001
	2	1030 (11)	558 (11)	472 (11)	
	3	5075 (53)	2741 (54)	2334 (52)	
	4	1868 (20)	1031 (20)	837 (19)	
N Stage	0	5788 (61)	3114 (62)	2674 (60)	0.06
	1	3065 (32)	1576 (31)	1489 (33)	
	2	656 (7)	361 (7)	295 (7)	
Grade	Well differentiated	1244 (13)	635 (13)	609 (14)	<0.0001
	Mod. differentiated	6648 (70)	3278 (65)	3370 (76)	
	Poorly Differentiated	1617 (17)	1138 (23)	479 (11)	
Age group	≤60	1925 (20)	798 (16)	1127 (25)	<0.0001
	61–70	2423 (25)	1189 (24)	1234 (28)	
	71–80	2814 (30)	1600 (32)	1214 (27)	
	>80	2347 (25)	1464 (29)	883 (20)	
Sex	Male	4913 (52)	2317 (46)	2596 (58)	<0.0001
	Female	4596 (48)	2734 (54)	1862 (42)	
Charlson Comorbidity Index	0	5957 (63)	3027 (60)	2930 (66)	<0.0001
	1–2	5083 (22)	1172 (23)	911 (20)	
	3–4	1023 (11)	596 (12)	427 (10)	
	5	446 (5)	256 (5)	190 (4)	
Adjuvant Chemotherapy	None	1775 (19)	955 (46)	820 (40)	0.0002
	Fluorouracil based	1098 (12)	553 (27)	545 (27)	
	Oxaliplatin doublet	1233 (13)	568 (27)	665 (33)	
	Unknown^a^	5403	2975	2428	
Year Diagnosed	2006–2009	5018 (53)	2644 (52)	2374 (53)	0.38
	2010–2013	4491 (47)	2407 (48)	2084 (47)	
Totals		9509	5051 (53)	4458 (47)	

^a^Not included in multivariate analysis in chemotherapy cohort

### 5 year all-cause mortality by primary tumour location

**Fig. 1:**
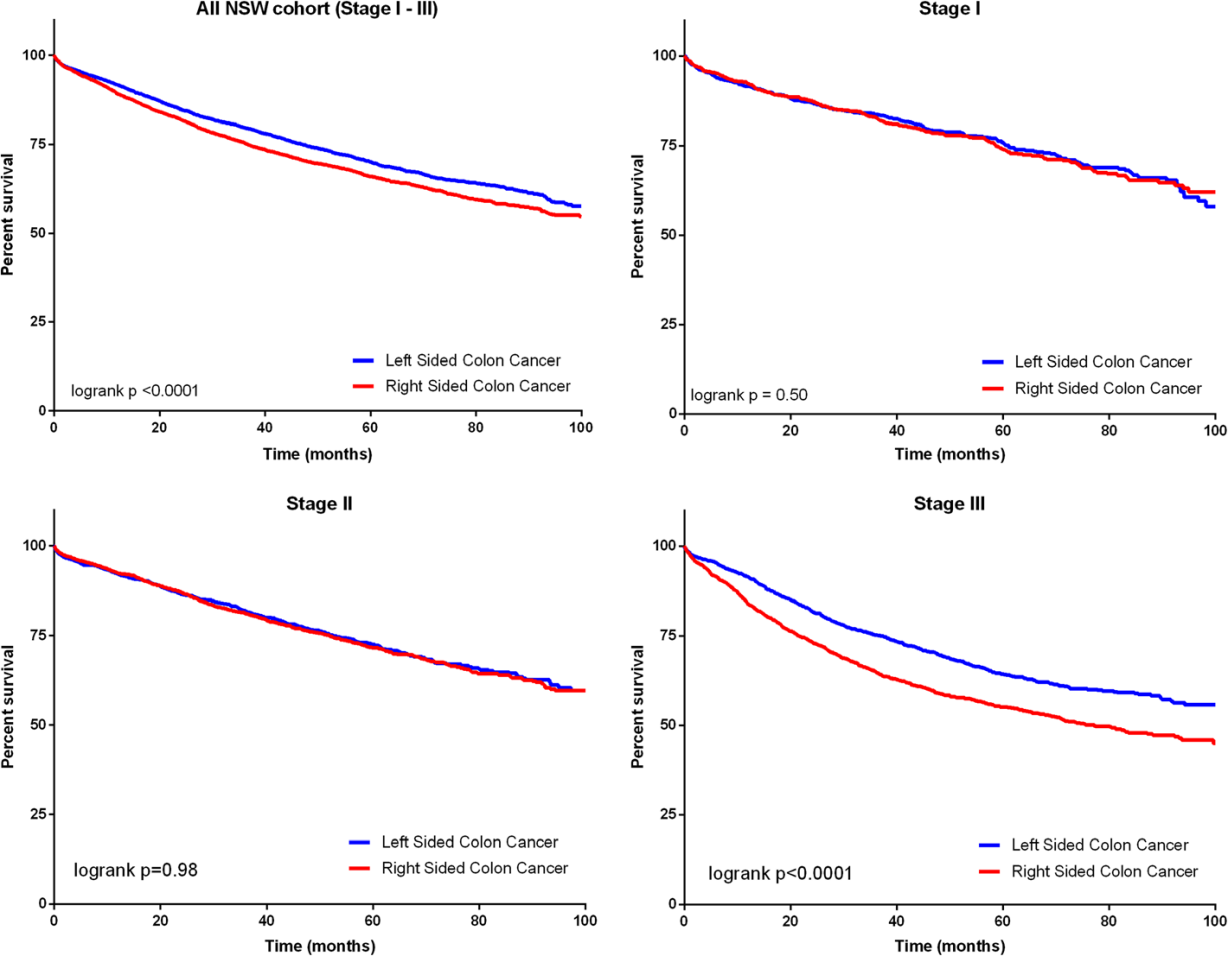
5 year all-cause mortality by primary tumour location *n* = 9509 patients with 2686 deaths (Stage *I* = 2104 patients with 440 deaths, Stage II = 3684 patients with 883 deaths, Stage III = 3721 patients with 1363 deaths)

The observed 5 year OS for patients with RsCC was 66% (95% CI 65–67%) compared to 70% (95% CI 69–72%) for LsCC. Unadjusted survival analysis demonstrated a higher mortality with RsCC in all stages combined (Fig. 1, univariate HR 1.20 95% CI 1.11–1.29, *p* < 0.0001). When stratified by stage there was significant difference in OS seen only in stage III, with a higher mortality seen in RsCC (Fig. 1, HR 1.46 95% CI 1.31–1.63, *p* < 0.0001) (Fig. 1).

After adjusting for sex, age, comorbidities, stage, grade, and year of diagnosis there was no significant difference in OS between RsCC and LsCC in patients from all stages (multivariate HR 0.96 95% CI 0.89–1.04 *p* = 0.35) (Table 2). When the multivariate analysis was stratified by stage, patients with RsCC had a trend to improved survival in stage I (HR 0.84 95% CI 0.69–1.01, *p* = 0.069), a statistically significant improved survival in stage II (HR 0.85 95% CI 0.75–0.98, *p* = 0.02), but a shorter survival in stage III (HR 1.13 95% CI 1.01–1.26, *p* = 0.03) (see Table 3.)

**Table 2:** Multivariate model for overall survival for NSW cohort (*n* = 9509)

Characteristic		Multivariate HR (95% CI)
Sided	Left	1
	Right	0.96 (0.89–1.04)
Age	≤60	1
	61–70	1.34 (1.15–1.56)
	71–80	2.23 (1.93–2.56)
	>80	3.97 (3.46–4.56)
Grade	Well differentiated	1
	Moderately differentiated	1.22 (1.06–1.39)
	Poorly Differentiated	1.87 (1.60–2.17)
TNM stage	I	1
	II	1.05 (0.96–1.21)
	III	2.00 (1.80–2.24)
Sex	Male	1
	Female	0.90 (0.83–0.97)
Charlson Comorbidity Index	0	1
	1–2	1.64 (1.49–1.79)
	3–4	1.81 (1.62–2.03)
	5	3.02 (2.63–3.46)
Year Diagnosed	2006–2009	1
	2010–2013	0.98 (0.90–1.06)

*HR* Hazard Ratio, *CI* confidence interval

**Table 3:** Univariate and multivariate Hazard Ratios for NSW cohort (*n* = 9509) stratified by stage. Statistically significant values in bold

		Overall Survival HR (95% CI)		Cancer Specific Survival HR (95% CI)	
		Univariate	Multivariate^a^	Univariate	Multivariate^a^
All Patients	Left Sided	**1**	1	1	**1**
	Right Sided	**1.20 (1.11–1.29)**	0.96 (0.89–1.04)	1.03 (0.91–1.18)	**0.84 (0.73–0.96)**
Stage I (*n* = 2104)	Left Sided	1	1	**1**	**1**
	Right Sided	1.03 (0.91–1.18)	0.84 (0.69–1.01)	**0.66 (0.45–0.95)**	**0.51 (0.35–0.75)**
Stage II (*n* = 3684)	Left Sided	1	**1**	**1**	**1**
	Right Sided	1.002 (0.88–1.14)	**0.85 (0.75–0.98)**	**0.68 (0.52–0.88)**	**0.59 (0.45–0.78)**
Stage III (*n* = 3721)	Left Sided	**1**	**1**	**1**	1
	Right Sided	**1.46 (1.31–1.63)**	**1.13 (1.01–1.26)**	**1.43 (1.21–1.69)**	1.12 (0.94–1.33)

^a^Following variables were used in the multivariate analysis: age, sex, year diagnosed, Charlson Comorbidity Index, TNM stage, grade

### Cancer specific survival (CSS) primary tumour location

**Fig. 2:**
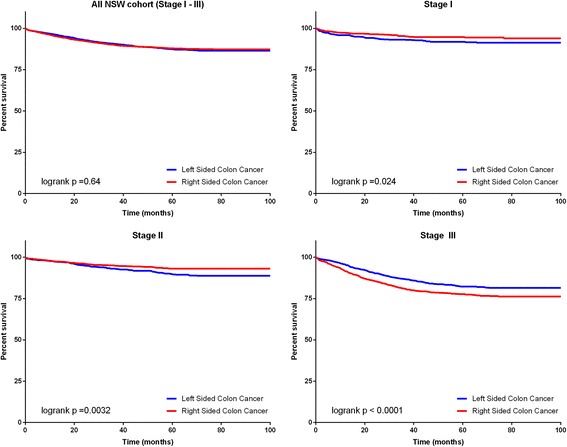
5 year cancer specific mortality by primary tumour location *n* = 9509 patients with 2686 deaths (Stage *I* = 2104 patients with 116 deaths, Stage II = 3684 patients with 224 deaths, Stage III = 3721 patients with 573 deaths)

The 5 year cancer specific survival (CSS) was similar for RsCC (89%; 95% CI 88–90%) and LsCC (89%; 95% CI 87–90%). Unadjusted CSS analysis did not show a significant difference between RsCC and LsCC in all stages combined (Fig. 2, univariate HR 1.03 95% CI 0.91–1.18, *p* = 0.64). When stratified by stage, there was a significantly improved CSS seen with RsCC in stage I (HR 0.66 95% CI 0.45–0.95, *p* = 0.024) and stage II (HR 0.68 95% CI 0.52–0.88 *p* = 0.0032), but a significantly poorer survival for stage III patients (HR 1.43 95% CI 1.21–1.66, *p* < 0.0001) (Fig. 2, Table 3).

In the multivariate analysis, after adjusting for sex, age, comorbidities, stage, grade, and year of diagnosis, patients with RsCC had a statistically significant improved CSS in all stages combined (HR 0.84, 95% CI 0.73–0.96, *p* = 0.011), and for stage I (HR 0.51 95% CI 0.35–0.75, *p* = 0.0006) and stage II (HR 0.59 95% CI 0.45–0.78, *p* = 0.0002) patients, but a trend to worse survival in stage III (HR 1.12 95% CI 0.94–1.33, *p* = 0.22) (Table 3).

### Effect of adjuvant chemotherapy

Adjuvant treatment details were available for 1631 (44%) of patients with stage II and 2441 (66%) of patients with stage III disease (4102 patients total). Most patients in stage II disease did not receive adjuvant chemotherapy (72%), with only a minority receiving fluorouracil monotherapy (24%) or an oxaliplatin doublet combination (usually FOLFOX, 5%). In contrast, the majority of patients with stage III disease received adjuvant chemotherapy (75%), with 28% treated with fluorouracil monotherapy, and 47% with an oxaliplatin/ fluorouracil doublet. Higher TNM-substage was associated with treatment with oxaliplatin doublet within both stage II (*p* < 0.0001) and III (*p* = 0.0001). Consistent with current practice no patients received adjuvant treatment with monoclonal antibodies.

Patients with RsCC were less likely to receive adjuvant chemotherapy (*p* = 0.0002, Table 1) despite higher risk tumour features. Adjuvant chemotherapy improved survival in both RsCC (univariate OS HR 0.68; 95% CI 0.58–0.80) and LsCC (univariate OS HR 0.48; 95% CI 0.40–0.58, Additional file 1: Figure S1 and Additional file 2: Figure S2.

Inclusion of the adjuvant chemotherapy regimen into the multivariate model did not alter the effect of primary tumour location, although the results for RsCC in stage II disease became non-significant (multivariate OS HR 0.86 95% CI 0.69–1.09 *p* = 0.19; multivariate CSS HR 0.67 95% CI 0.43–1.04, *p* = 0.07, Table 4). Patients with RsCC in stage III colon cancer continued to have a significantly inferior OS compared to LsCC even after adjustment for all above factors including receipt and type of adjuvant chemotherapy (multivariate OS HR 1.29 95% CI 1.11–1.50 *p* = 0.0012; multivariate CSS HR 1.16 95% CI 0.92–1.47, *p* = 0.22, Table 4). When analyses were restricted to only those stage III patients who received adjuvant oxaliplatin doublet chemotherapy (*n* = 1233), RsCC remained associated with a poorer OS (univariate OS HR 1.8 95% CI 1.4–2.4, *p* < 0.0001).

**Table 4:** Multivariate model for overall survival for chemotherapy cohort (*n* = 4102)

Characteristic		Stage II (*n* = 1631)	Stage III (*n* = 2441)
		Multivariate	Multivariate
		HR (95% CI)	HR (95% CI)
Sided	Left	1	1
	Right	0.86 (0.68–1.09)	1.29 (1.11–1.50)
Age	≤60	1	1
	61–70	1.90 (1.20–2.99)	1.21 (0.94–1.54)
	71–80	2.97 (1.92–4.58)	1.81 (1.43–2.30)
	>80	5.92 (3.82–9.19)	2.00 (1.54–2.60)
Grade	Well/mod differentiated	1	1
	Poorly Differentiated	1.43 (1.08–1.90)	1.49 (1.26–1.75)
TNM stage	IIIa	1	1
	IIIb	2.20 (1.71–2.82)^a^	1.79 (1.33–2.43)
	IIIc	-	3.86 (2.84–5.24)
Sex	Male	1	1
	Female	0.85 (0.68–1.07)	0.94 (0.82–1.10)
CCI	0	1	1
	1–2	1.42 (1.09–1.52)	1.15 (0.96–1.38)
	3–4	1.60 (1.12–2.28)	1.20 (0.94–1.53)
	5	2.31 (1.45–3.69)	1.83 (1.36–2.46)
Year Diagnosed	2006–2009	1	1
	2010–2013	0.99 (0.79–1.26)	1.00 (0.86–1.17)
Adjuvant Chemotherapy	Nil	1	1
	Fluorouracil monotherapy	0.79 (0.51–1.10)^b^	0.48 (0.40–0.57)
	Oxaliplatin doublet	-	0.38 (0.27–0.42)

*HR* Hazard Ratio, *CI* confidence interval, *CCI* Charlson Comorbity index

^a^IIa vs IIb/IIc, ^b^chemotherapy vs no chemotherapy

## Discussion

There are well established differences in patient demographics, tumour factors and clinical presentation between RsCC and LsCC [7, 9, 10, 17, 18]. However it remains uncertain whether primary tumour location is an independent prognostic factor in locoregional colon cancer.

The strongest evidence comes from a recent meta-analysis of 66 studies including 1,437,846 patients which showed LsCC is associated with a significantly reduced risk of death compared to RsCC (HR 0.82; 95% CI 0.79–0.84, *P* < 0.01) [12]. This study included all stages of colon cancer and found that, based on meta-regression, the effect of primary tumour location was independent of stage, race, year of study, and quality of study.

It is important to consider the limitations of the above meta-analysis. Firstly, there was significant heterogeneity seen in the results (I^2^ = 93%), which is likely due to the variety of included study designs, differing multivariate covariates from source studies, and patient populations, with the estimate derived from overall populations with no stratification by stage.

Secondly, while most of the included studies controlled for tumour factors (such as stage and grade), and patient demographic factors (eg., age, sex), only three studies included a comorbidity index in the multivariate model [7, 17, 19], and only 21% (14 of 66 studies) included performance status. RsCC is more likely to occur in older patients who have more associated comorbidities [17], and the substantial imbalances in the baseline characteristics between LsCC and RsCC patients in these trials may be an unmeasured confounder which explains the improved survival with LsCC. This issue has been directly addressed by Warschkow et al. [9] who, in order to minimise confounding, used propensity score matching to analyse survival in RsCC versus LsCC in 91,416 patients with stage I-III colon cancer from the SEER database. These authors showed that RsCC had a better OS (HR 0.89, *p* < 0.001) and CSS (HR 0.71, *p* < 0.001) in stage I and II, but a similar prognosis in stage III (OS HR 0.99, *p* = 0.49; CSS HR 1.04, *p* = 0.129).

Our current study, using a large series of Australian patients from a prospectively collected database, and controlling for patient factors (including comorbidities), tumour factors, and adjuvant chemotherapy, confirmed previous studies showing that RsCCs are more likely to have a more advanced stage (*p* < 0.0001) and grade (*p* < 0.0001), and occur in older patients (*p* < 0.0001) with more comorbidities (*p* < 0.0001). Despite higher risk tumour features, patients with RsCC are less likely to receive adjuvant chemotherapy (*p* < 0.0001) or oxaliplatin doublet chemotherapy (*p* = 0.0002).

In the survival analysis, patients with RsCC have a lower all-cause mortality in stage II (HR 0.85, *p* = 0.02), but a higher mortality in stage III (HR 1.13, *p* = 0.032). Moreover, patients with RsCC had an improved 5-year CSS in Stage I (HR 0.51,*p* = 0.0006) and Stage II (HR 0.59, *p* = 0.0002), and a trend to inferior CSS in Stage III.

As adjuvant chemotherapy has been shown to have a larger benefit in RsCC than LsCC [20], we subsequently undertook further multivariate analysis in a subset of patients with known adjuvant chemotherapy protocols to validate our findings. Adjuvant chemotherapy improved survival in both RsCC and LsCC. We found incorporation of adjuvant chemotherapy into the multivariate model did not alter the effect of primary tumour location. Although definitive conclusions were limited in stage II as chemotherapy regimens where only available in 44% of patients, there were similar hazard ratios showing improved OS and CSS with RsCC (multivariate HR 0.86 and 0.67 respectively), although statistically non-significant in the chemotherapy cohort. In stage III, where chemotherapy data was available for the majority of patients (66%), the results of multivariate analysis was very similar to overall cohort, with a significantly higher all-cause mortality with RsCC (HR 1.29, *p* = 0.0012) and trend to higher cancer specific mortality (HR 1.16, *p* = 0.21).

Our findings are consistent with the results of Wiess et al. [7], a large multivariate retrospective analysis of 53,801 patients from the SEER database linked to Medicare data, and controlled for comorbidities using Hierarchical Condition Categories risk score. Similar to our findings, in multivariate analysis, patients with RsCC had a non-significant trend to lower mortality in stage I (HR 0.95, *p* = 0.21), a lower mortality in stage II (HR 0.92, *p* < 0.0001), but a higher mortality in stage III (HR 1.12, *p* < 0.001), and a non-significant difference in mortality overall (HR 1.01, *p* = 0.60). This stage dependant effect, with an improved survival in RsCC in stage II, but higher mortality in stage III, has been reported by multiple other series [8–10, 18, 21].

The cause of the demonstrated inconsistent effect of primary tumour location by stage is unclear. Our study, and the quoted literature, are retrospective analyses of large population databases, and are susceptible to the inherent bias of confounding associated with this study design. However an alternative explanation to consider is the increasingly described differences in tumour biology between RsCC and LsCC. RsCCs are more likely to have adverse histological features (such as advanced T stage, higher grade, or lymophvascular invasion) and mucinous histology [2, 22–24]. Perhaps more importantly, there are also marked differences in the molecular profile between these tumours [25]. RsCC has a higher rate of BRAF mutations and high microsatellite instability (MSI-H), both which have established prognostic importance, with MSI-H tumours shown to have a favourable prognosis, and BRAF a strong poor prognostic marker in non-MSI-H but not in MSI-H tumours [22, 23, 26, 27]. In addition even within MSI-H tumours there are known differences in prognosis, with hereditary MSI-H colon cancers shown to have a better survival than sporadic cases [28]. It is important to note that these biomarkers are not uniformly distributed by stage, with MSI-H tumours associated with lower stage (21% in stage II vs 14% stage III and 4% stage IV), and BRAF mutant tumours more likely to occur at a higher stage [22, 29, 30]. Furthermore, previous studies have shown a differential effect of adjuvant chemotherapy in between molecular subtypes. There is a reduced benefit with fluorouracil based chemotherapy in MSI-H tumours, but preserved efficacy of oxaliplatin in MSI-H stage III colon cancer patients [31, 32]. Although our study demonstrated a persistent effect of primary tumour location even when OS analysis was restricted to those patients who received adjuvant oxaliplatin doublet chemotherapy, it is important to note that fewer patients with RsCC received oxaliplatin as part of the adjuvant treatment.

Therefore, in the absence of both family history and molecular profiles in these population series, it is reasonable to hypothesise that some of the observed survival difference in stage II and III may be due to unequal distribution of these biomarkers. However, emerging evidence suggests that primary tumour location may be a clinical surrogate for further, yet unidentified, predictive biomarkers as highlighted by the recent data from the FIRE3 and CALGB/SWOG 80405 trials, which suggests a reduced benefit to anti-EGFR treatment in RsCC independent of currently identified biomarkers [33]. A limitation of our study is the lack of associated molecular data which is a potential source of unmeasured confounding to the results.

## Conclusion

This population based study provides further evidence that primary tumour location is an important independent clinical prognostic factor in stage II and III colon cancer with immediate implications for clinical practice and trial design. This clinical biomarker is likely acting as a surrogate for as yet unidentified molecular factors. Further studies with associated tumour molecular profiles are required to clarify the underlying biological differences between RsCC and LsCC.

## Additional files

Additional file 1: Figure S1. Effect of adjuvant chemotherapy on overall survival in patients with right sided colon cancer. Description: Overall survival in patients with right sided colon cancer by receipt of adjuvant chemotherapy (*n* = 2076). (TIFF 40 kb)

Additional file 2: Figure S2. Effect of adjuvant chemotherapy on overall survival in patients with left sided colon cancer. Description: Overall survival in patients with left sided colon cancer by receipt of adjuvant chemotherapy (*n* = 2030). (TIFF 40 kb)

## Abbreviations

CSS: Cancer specific survival
HR 95% CI: Hazard ratios and 95% confidence intervals
LsCC: Left sided colon cancer
MSI-H: High microsatellite instability
NSW: New South Wales
OS: Overall survival
RsCC: Right sided colon cancer
